# Full coverage path planning algorithm for MRgFUS therapy

**DOI:** 10.1002/rcs.2389

**Published:** 2022-03-13

**Authors:** Anastasia Antoniou, Andreas Georgiou, Nikolas Evripidou, Christakis Damianou

**Affiliations:** ^1^ Department of Electrical Engineering Computer Engineering, and Informatics Cyprus University of Technology Limassol Cyprus

**Keywords:** algorithm, MRgFUS, path planning, plastic films, segmentation

## Abstract

**Background:**

High‐quality methods for Magnetic Resonance guided Focussed Ultrasound (MRgFUS) therapy planning are needed for safe and efficient clinical practices. Herein, an algorithm for full coverage path planning based on preoperative MR images is presented.

**Methods:**

The software functionalities of an MRgFUS robotic system were enhanced by implementing the developed algorithm. The algorithm's performance in accurate path planning following a Zig‐Zag pathway was assessed on MR images. The planned sonication paths were performed on acrylic films using the robotic system carrying a 2.75 MHz single element transducer.

**Results:**

Ablation patterns were successfully planned on MR images and produced on acrylic films by overlapping lesions with excellent match between the planned and experimental lesion shapes.

**Conclusions:**

The advanced software was proven efficient in planning and executing full ablation of any segmented target. The reliability of the algorithm could be enhanced through the development of a fully automated segmentation procedure.

## 1 INTRODUCTION

High Intensity Focussed Ultrasound (HIFU) has been used in the clinical management of various medical diseases as a beneficial non‐invasive alternative to traditional therapeutic approaches.^1^ The HIFU‐induced thermal effects have been extensively exploited in oncology, where the ultrasound beams are concentrated into a mm‐sized location causing rapid temperature elevation in local tissue, without damaging tissues in the acoustic path.^1^ Exposure of tissue to temperatures higher than 60°C for 1 s was proven sufficient to produce coagulation necrosis and lesion formation.^2^ Since tumours have a diameter of several centimetres, ablation of their entire volume requires generating lesions side‐by‐side. Navigation of the beam focus through the entire tumour is achieved by mechanically moving the source or by electronically steering the beam using phased array technology.^3^

Through the view of physicians, high‐quality methods for treatment planning and real‐time monitoring of heating during HIFU are needed for safe and efficient clinical practices. Briefly, the planning process involves pretherapy imaging and tumour segmentation followed by administration of sonication points throughout the marked region of interest (ROI) and selection of the proper scanning pathway.^4^ The sonication protocol is then executed commonly under the guidance of ultrasound (US) or Magnetic Resonance Imaging (MRI).^1^ Although both constitute well‐established non‐invasive imaging modalities, MRI produces anatomical images of significantly higher resolution regardless of depth and intervening structures, thus enabling tissue targeting and focus positioning with very high precision.^5^ Simultaneously, MRI has the unique ability of intraoperative temperature monitoring for selective tissue ablation through MR‐based thermometry.^6^ The HIFU technology combined with MRI is known as Magnetic Resonance guided Focussed Ultrasound (MRgFUS).

The first step in the planning process is localising the ROI in preoperative MR images.^4^ Currently, localisation of the ROI typically requires manual involvement by a trained physician, which unavoidably decreases the therapeutic efficacy.^7^ Clinical practices will thus be greatly benefited by computer‐based methods for automated segmentation and full coverage path planning.^4^ As an example, Loeve et al.^4^ reported an average time of 18 min for image segmentation of the target based on observations of MRgFUS interventions in the uterine, suggesting that the total treatment duration could be decreased by automated segmentation, especially when multiple segmentation adjustments are needed for motion compensation.^4^

Since MRI is routinely employed in brain disease detection,^8^ there is a wide variety of techniques for brain lesion segmentation in MR images available in the literature, which may be helpful given the adaption of MRgFUS as a neurotherapeutic tool.^9^ Zhang et al.^10^ classified the existing methods into three main categories; conventional methods (i.e., threshold, region, fuzzy theory, and edge detection), classical machine learning‐based methods (i.e., K Nearest Neighbour ‐ KNN, random forest, Contingent Valuation Method ‐ CVM, and dictionary learning) and deep learning‐based methods (i.e., Convolutional Neural Network ‐ CNN, Fully Convolutional Network ‐ FCN, and encoder‐decoder). Another similar review study^11^ concluded that the tumour segmentation is more effective when a fully CNN is combined with a Conditional Random Field (CRF) statistical method.

The‐wide adaption of HIFU in the clinical management of uterine fibroids has led scientists to the development of more advanced planning and guiding tools for this specific application. Xu et al.^12^ proposed an automatic segmentation method for uterine fibroids on US images. The developed algorithm involves dividing the image into smaller regions called superpixels that are characterised using a texture histogram‐based feature representation method and finally merging them again based on their similarities, meaning that the tumour's pixels will be merged together since they have similar texture.^12^ Recently, Ning et al.^7^ proposed an image guidance system featuring tools for automatic detection and segmentation of lesions on MR images through a CNN multi‐stage segmentation. Notably, the developed system also enables intraoperative lesion tracking on US images.^7^

Recently, in the effort to enhance MRgFUS therapy planning, three classical methodology types were assessed for their performance in segmenting ROIs (e.g., tissue, water, and transducer) in MR images of a HIFU setup.^13^ The tested methods were the simplest image segmentation method known as the Threshold method, the Watershed segmentation algorithm with markers (WSAM), and two Level set methods (LSM); the Geodesic Active Contours (GAC) and the Distance Regularised Level Set Evolution (DRLSE) methods. Preliminary results were promising; however, the methods are accompanied by some limitations, such as the need to establish an initial contour for GAC and DRLSE methods and the complex procedure of defining the markers of WSAM, which both require previous intervention by the user for MR image division.^13^

The segmentation procedure is followed by path planning for selective ablation of the delineated ROI.^4^ Selection of the proper scanning pathway is essential in forming uniform lesions throughout the segmented target and minimising thermal exposure of normal tissue.^14^ A conventional scanning approach in clinical HIFU is the Raster scanning where the ultrasonic source sequentially visits spots arranged in horizontal lines that are scanned in the same direction.^14^ Thermal diffusion was proven an essential phenomenon reducing the therapeutic outcome of this scanning mode through the formation of asymmetric lesions.^14^, ^15^, ^16^ This is attributed to that lesion formation at a specific spot is affected by the thermal energy diffusing from neighbouring previously sonicated spots, thereby leading to inadequate treatment of initial spots and extensive heating of the later ones, as well as excess thermal dose deposition in the pre‐focal area, a phenomenon known as the near‐field heating.^17^

Therefore, there are still challenges in achieving ablation of a well‐defined area by eliminating thermal diffusion effects while ideally using the minimum energy and treatment time possible. In this effort, researchers have investigated how various scanning paths and the used sonication parameters affect the therapeutic result of HIFU therapy.^14^, ^18^, ^19^, ^20^, ^21^ Zhou et al.^14^ investigated how the Spiral scanning from the centre to the outside and vice versa affect the formation of lesions by sonicating a gel phantom and bovine liver in a discrete rhombus‐shaped grid. The proposed scanning approaches produced more uniform lesions but of a smaller volume than conventional raster scanning when used under the same protocol.^14^ Qian et al.^18^ also investigated the performance of a Spiral pathway that was executed in a continuous scanning mode covering a square area in acrylamide‐based heat‐sensitive phantom and bovine liver. The results suggest that uniform lesions without overheating phenomena can be produced by continuous scanning along the spiral pathway by selecting proper scanning speed to regulate thermal energy diffusion.^18^

Typically, discrete scanning modes employ a time delay between successive sonications of equal duration to eliminate intense heating.^17^ In accordance, the accumulation of thermal energy should be controlled by selecting not only the proper pathway, but also sufficient cooling periods. A recent study^17^ evaluated the effect of increasing time delay on the induced near‐field heating and overall treatment time for six different pathway algorithms, including the commonly used Sequential and Spiral algorithms. It is noted that the Sequential algorithm differs from the aforementioned Raster scanning only in that adjacent lines are scanned in opposite directions. Experimental evaluation in a tissue‐mimicking phantom (TMP) revealed that a minimum time delay of 50–60 s is needed for achieving a safe thermal dose in the near‐field region.^17^

At this point, it is interesting to note that the use of different sonication times at the various grid spots was proposed as an alternative method to avoid the introduction of cooling intervals, thus minimising the overall treatment time.^19^ The relevant article compared the performance of the Raster, Spiral, and Skip paths using unequal heating duration with the conventional scanning mode by simulating the ablation of a square area through electronic steering of the beam according to each pathway.^19^ The results suggest that the proposed method is robust when used in combination with the Skip scanning path and could lead to a treatment time reduction of more than 50%.^19^

Herein, a full area coverage path planning algorithm intended for planning MRgFUS sonication protocols is presented. The ROI in a medical image is initially marked following placement of indicative points by the user. In brief, sonication points are then placed throughout the segmented ROI and sorted based on a Zig Zag pathway to be sequentially visited by the ultrasonic source.

The accuracy of the developed algorithm was evaluated using an MRgFUS robotic system intended for preclinical use. The algorithm was implemented within the relevant controlling software. Once the power field of the incorporated transducer was tested ensuring sufficient emission of ultrasonic energy, the performance of the algorithm was assessed by path planning on medical images and execution of the planned sonications on plastic films for visual assessment of accuracy.

Advantageously, the proposed algorithm is specifically dedicated to path planning of MRgFUS robotic devices, whereas authors in previous studies developed planning tools for US‐guided therapy^7^, ^12^ or examined the performance of already existing segmentation tools.^13^ With the proposed algorithm, the whole procedure from the lesion segmentation stage and distribution of foci to path execution is implemented in the MRI setting. In addition, the algorithm is simplified and fast, and thus more ergonomic in its use.

## 2 MATERIALS AND METHODS

### 2.1 Robotic system for MRgFUS preclinical use

A robotic system intended for preclinical applications of MRgFUS was used for the purposes of the study.^22^ The system was manufactured on a rapid prototyping machine (FDM400, Stratasys, 7665 Commerce Way, Eden Prairie, Minnesota, 55344, USA) with Acrylonitrile Butadiene Styrene (ABS) thermoplastic. All the components were selected based on MR compatibility to enable proper operation in the MRI environment. Due to its compact design (57 cm in length, 21 cm in width and 11.5 cm in height) is capable to be fitted inside the MRI operating table for prone positioning of the subject, simultaneously facilitating ease of transfer, also given its lightweight design of about 5 kg. Accordingly, the treatment area is targeted from the bottom to the top.

**FIGURE 1:**
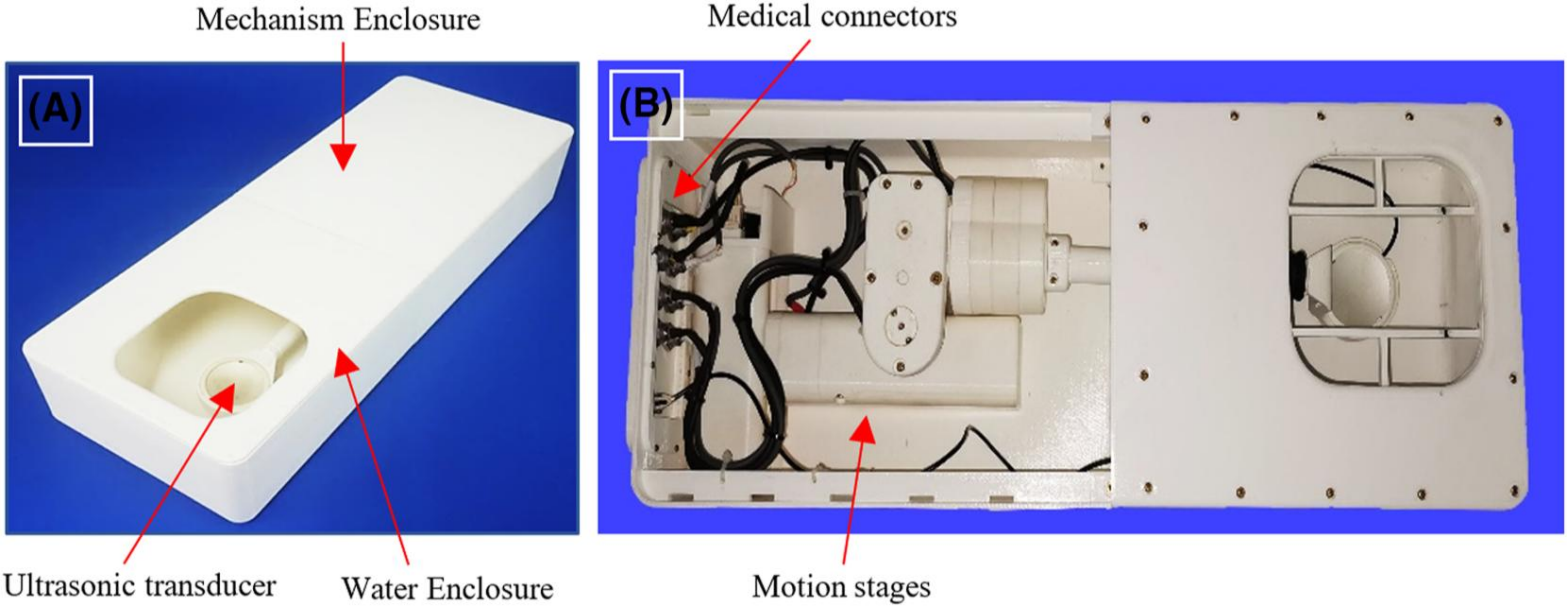
(A) Exterior and (B) interior views of the 4 degrees of freedom (DOF) Magnetic Resonance guided Focussed Ultrasound (MRgFUS) robotic device

The main device comprises an enclosure where the motion mechanism is actuated, being driven by piezoelectric motors (USR30‐S3, Shinsei Kogyo Corp., Tokyo, Japan) in 4 computer‐controlled degrees of freedom (DOF) for positioning the ultrasonic transducer about the subject. The transducer is accommodated in a separate water‐filled enclosure through an arm extending from the mechanism. In this study, a single element spherically focussed transducer with a nominal frequency of 2.75 MHz, a radius of curvature of 65 mm, and a diameter of 50 mm was integrated with the system. The transducer was tuned with an RF amplifier (AG1016, AG Series Amplifier, T & C Power Conversion, Inc., Rochester, US) for optimum power output, achieving a percent acoustic efficiency of 30%. Notably, the positioning mechanism is robust enough to carry a phased array transducer. The described system is shown in Figure 1.

### 2.2 Software for robotic control

**FIGURE 2:**
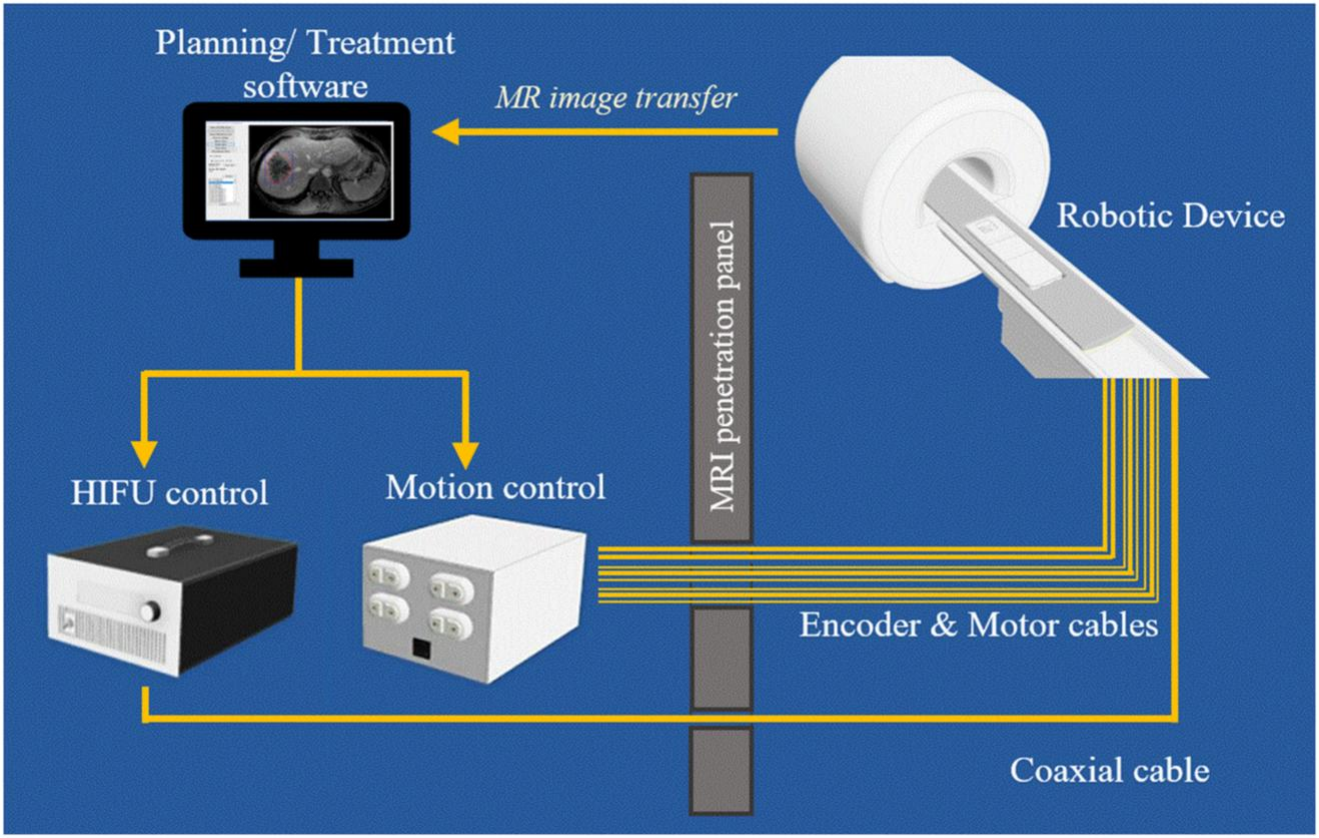
Block diagram of the connection between hardware and software through the Magnetic Resonance Imaging (MRI) penetration panel

The various functionalities of the system are controlled remotely through an in‐house developed software written in C sharp programing language (Visual Studio 2010 Express, Microsoft Corporation). The main algorithms implemented allow for controlling the robotic motion and FUS operation. The user commands motion in grid patterns by specifying the relevant parameters (grid size, motion pattern, spatial step, and time delay between successive sonications) and visualizes the grid operation in real time through a user‐friendly graphical interface. The sonication parameters at each spot are easily adjusted by the user. This software can be interfaced with the MRI so that medical images are directly transferred for further processing and treatment planning, as well as for controlled ultrasonic exposures through the use of MR thermometry.^6^ Figure 2 shows a block diagram of the connection between hardware and software through the MRI penetration panel.

### 2.3 Path planning algorithm

**FIGURE 3:**
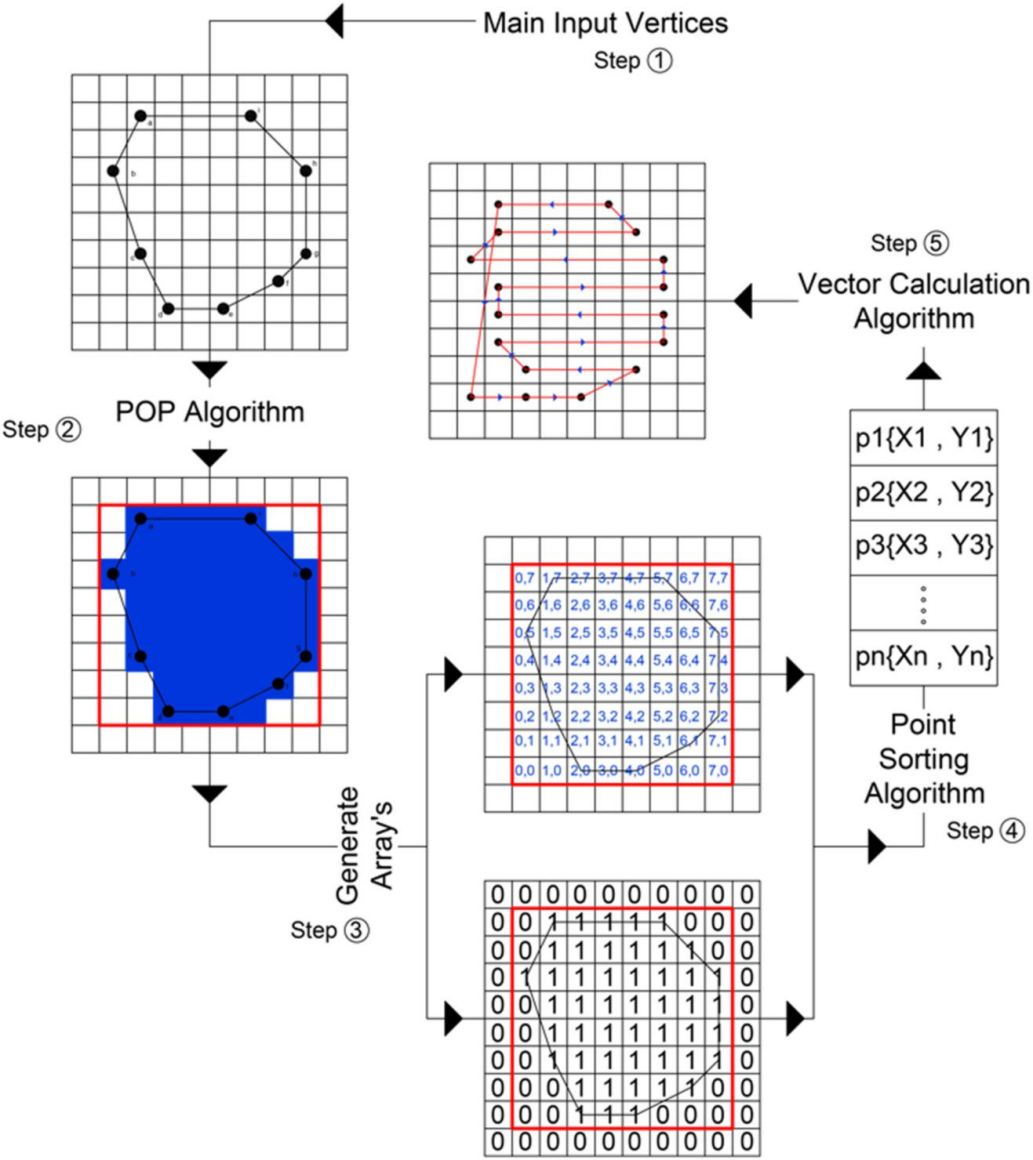
Schematic diagram of the developed algorithm showing the flow between the 5 subprocesses

**FIGURE 4:**
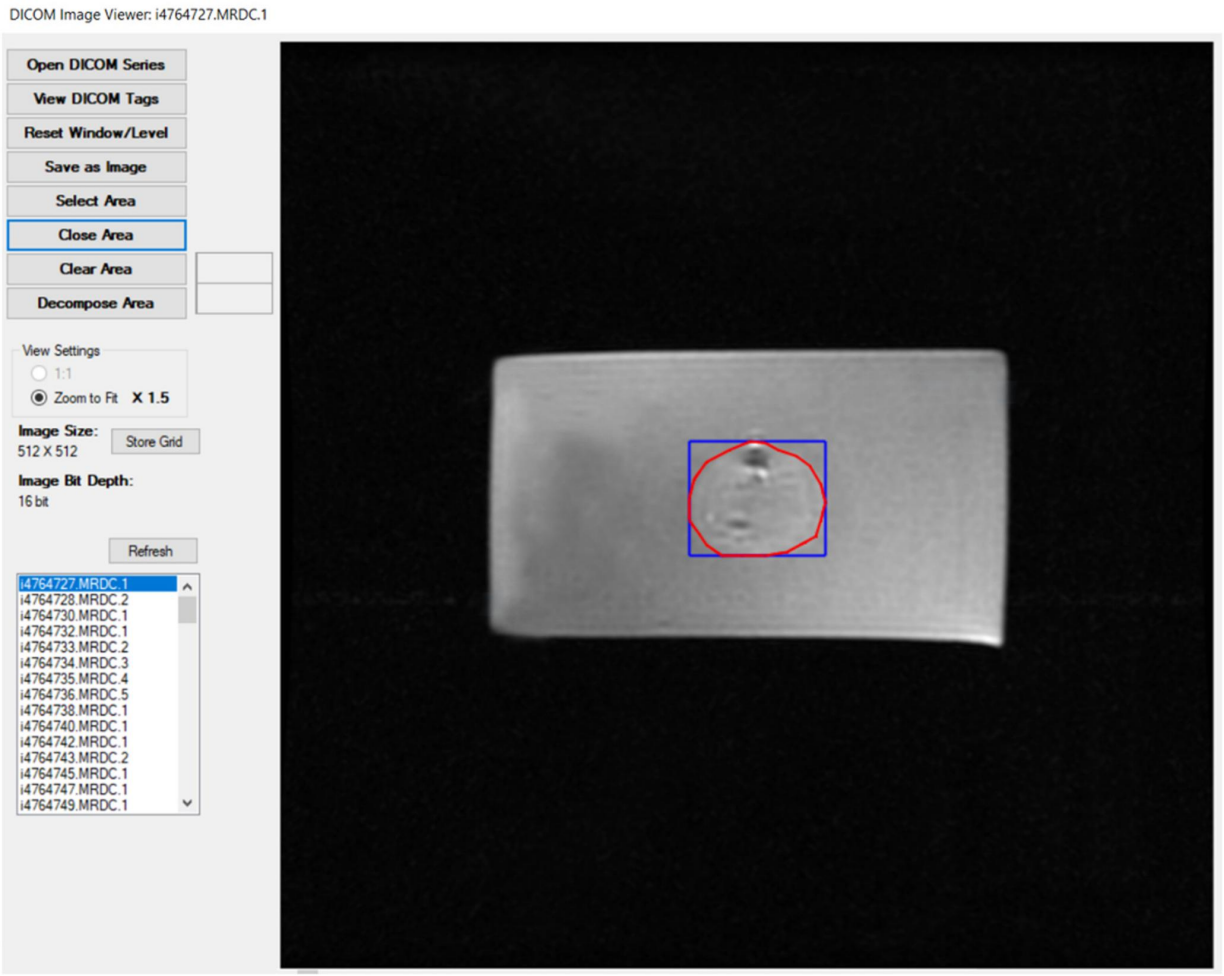
Screenshot of the software interface for path planning

The software was extended with additional functionalities to support therapy planning. The developed algorithm consists of 5 basic subprocesses as illustrated in the schematic diagram of Figure 3. The algorithm receives as input a list of vertices (at least 3) that are manually selected by the user as points (x, y) on the medical image from the graphical user interface of the software. These points are connected forming a polygon indicating the ROI where sonication will take place. The user should also select the intended motion step in mm for the X and Y axes, which defines the resolution of sonication. The second subprocess involves the Point of Polygon (POP) Algorithm that is responsible for identifying which pixels of the entire medical image are included within the delineated area based on the Jordan Curve Theorem.^23^ Once the involved pixels are identified, a boundary box is created around the segmented area. Binary arrays are generated so that each pixel is assigned a number (0 or 1) defining whether or not it should be sonicated. The localised sonication points are then sorted in the order to be visited by the transducer based on a Zig Zag pattern (step 4). Finally, the sorted points are converted into motion vectors through step 5. A homing procedure follows where the offset between the origin point of the sonication frame (boundary box of the segmented area) and the real location of the transducer is compensated. The extracted path planning vectors serve as the output defining the transducer's path in two‐dimensional space for full coverage of the segmented area. These motion vectors are sent successively to the driving system for execution. Figure 4 is a screenshot of the software interface for path planning.

### 2.4 Evaluation of power field

The power field of the transducer was evaluated by HIFU sonications on clear films made of acrylic plastic with a thickness of 0.9 mm (FDM400mc print plate, Stratasys). The ultrasonic attenuation of the plastic films was estimated at 8.5 dB/cm‐MHz (at 2 MHz) according to the transmission through technique.^24^ The focussed transducer was mounted on a special holder that was geometrically designed to enable horizontal placement of the film at varying distance above the transducer and the whole structure was immersed in degassed water for proper ultrasonic transmission. The films were sonicated at various distances from the transducer's surface using a constant electric power of 150 W (acoustic power of 45 W) and a sonication time of 10 s. The upper side of the film involved air, and thus, the heating of the film was mainly based on reflection. The diameter of the formed lesion at each distance was estimated.

### 2.5 Evaluation of the algorithm's performance

The accuracy of the proposed algorithm was evaluated by path planning on medical images. MR images of brain and TMPs were imported in the software for processing by the user. The sonication protocols were automatically developed after placement of initial points by the user and executed on acrylic films to assess the performance of the algorithm as implemented in the FUS procedure. Simultaneously, the robotic system was evaluated in terms of proper communication with the software and accuracy of robotic motion.

**FIGURE 5:**
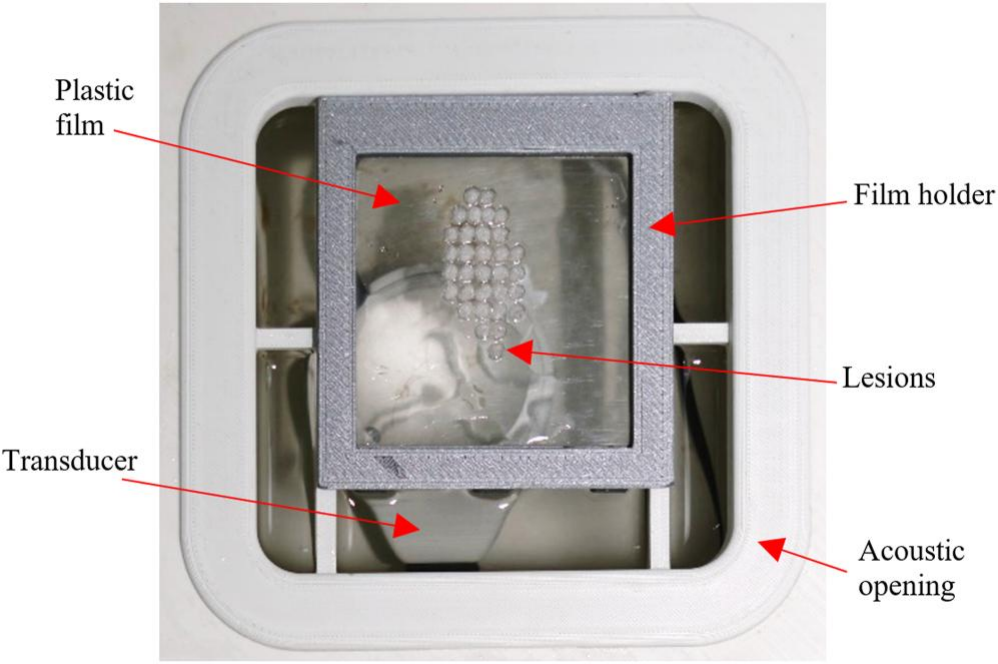
Photo of the experimental setup used for sonicating plastic films

Plastic films with approximate dimensions of 82 × 87 × 0.9 mm were fixed to the acoustic opening of the water enclosure at a constant distance of approximately 55 mm above the transducer's surface with the assistance of a special holder as illustrated in Figure 5. Again, degassed water served as the coupling medium. Multiple sonications along the planned Zig‐Zag pathway were performed using grid operation with a 2 mm step. Each grid spot was sonicated using a moderate acoustical power of 30 W for 7 s with a time delay of 30 s between successive expositions. It is noted that the optimum sonication time for lesion formation was selected at 7 s following initial testing with varying sonication times. The selection of a proper time delay was mainly based on the results of a previous study of the group^17^ suggesting that a time delay of 60 s is needed between 2 mm spaced sonication spots to reduce off‐target heating in an agar‐based TMP. Given that thermal diffusion is a less effective mechanism of energy loss in plastic, a 30 s delay was considered sufficient.

## 3 RESULTS

**FIGURE 6:**
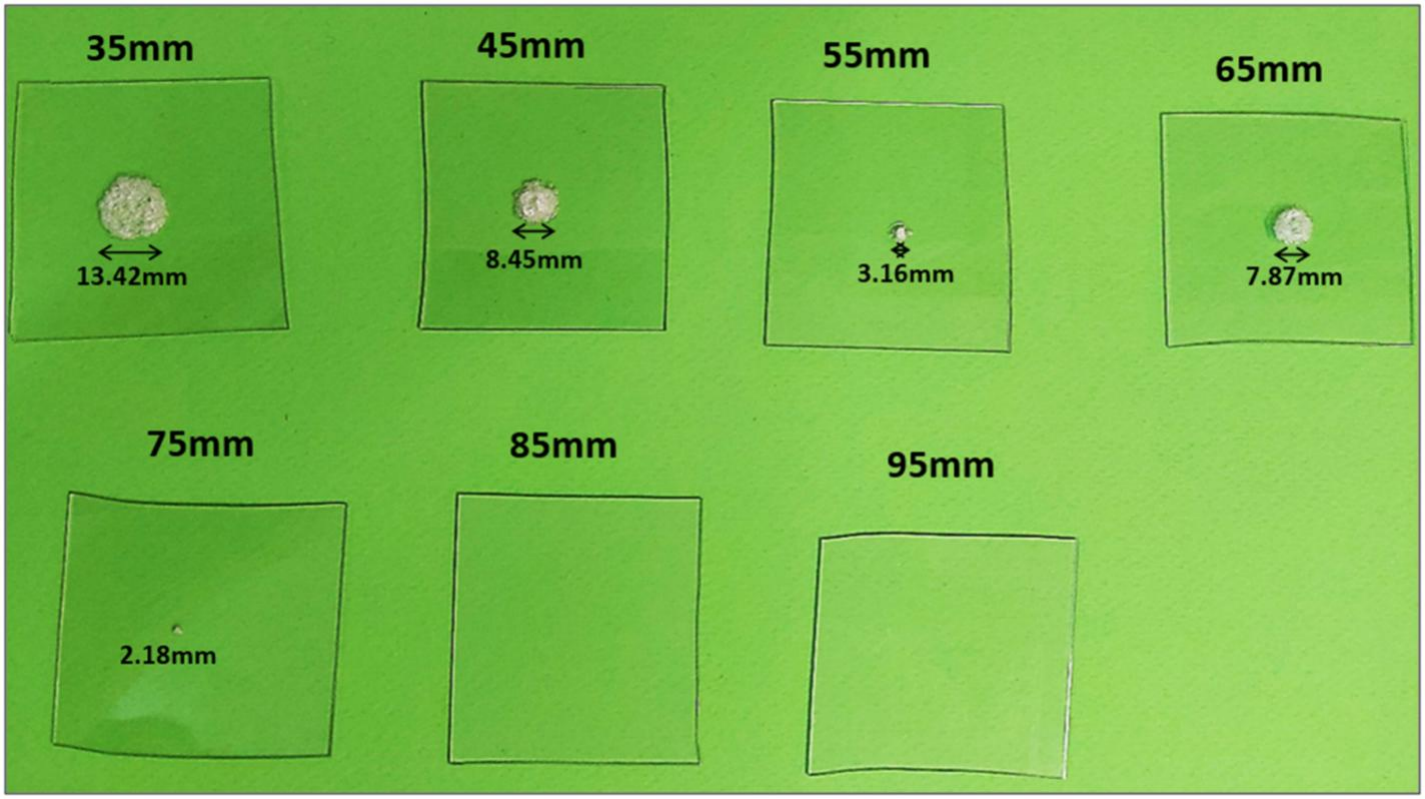
Photo of films sonicated at increasing depth from the surface of a single element transducer with central frequency of 2.75 MHz, radius of curvature of 65 mm, and diameter of 50 mm, indicating the diameter of the formed lesions

An indication of the power field of the ultrasonic transducer was obtained by sonicating plastic films at increasing depth of 35–95 mm from the transducer's surface. Figure 6 shows a photo of the sonicated films indicating the diameter of the formed lesions. Visual assessment reveals a focal spot shifted at 55 mm. Notably, large lesions were observed at distances of 35, 45 and 65 mm indicating increased heating in the near‐field region. No lesions were observed at distances of 85 and 95 mm. The change in the lesion size with varying distance is indicative of the power field distribution. Specifically, assuming a Gaussian distribution of acoustic power around the maxima in axial and radial planes, the dimensions of the formed lesions are defined by the half power length and width, respectively.

**FIGURE 7:**
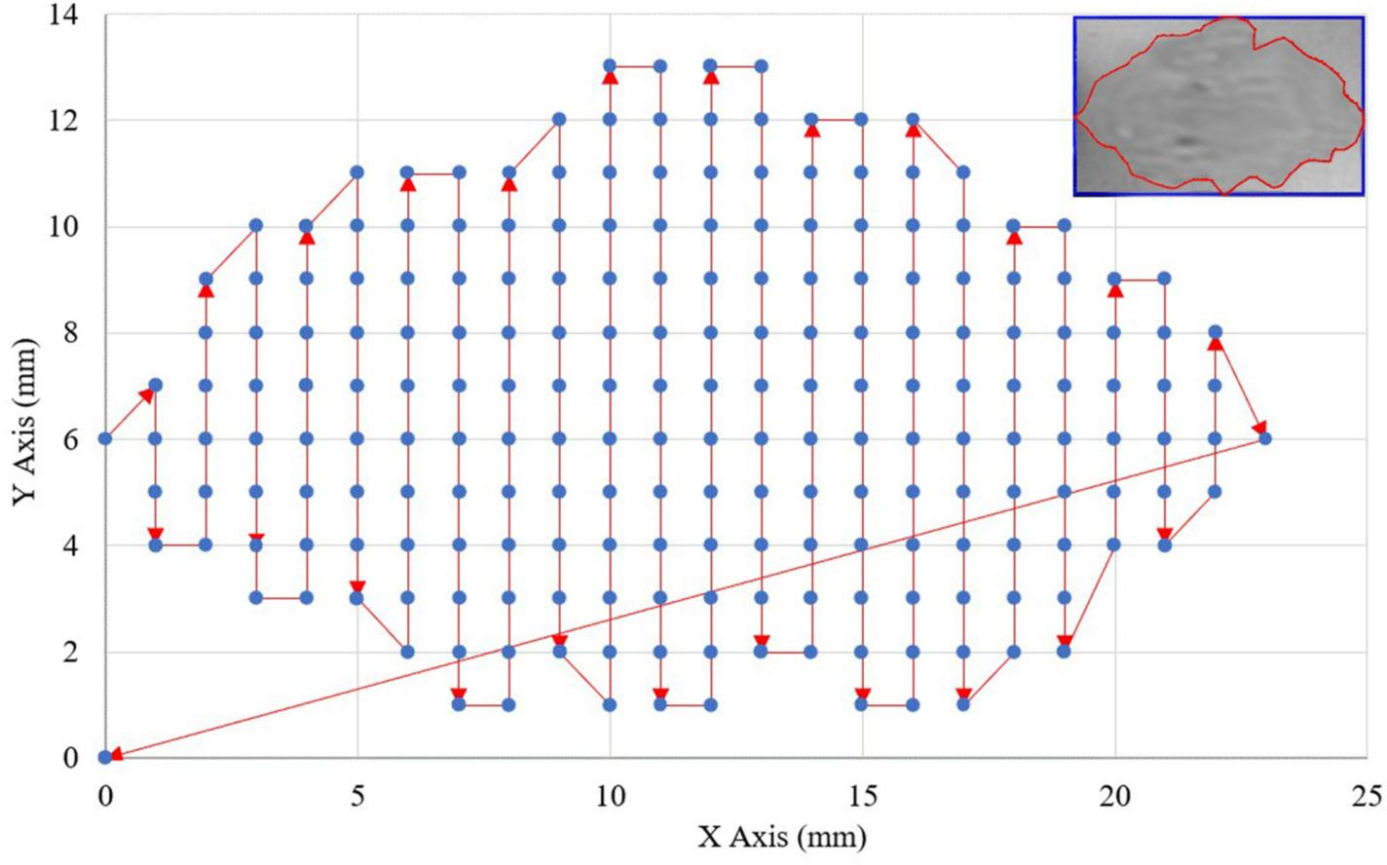
Representation of sonication spots arranged in the X‐Y plane and the planned Zig‐Zag pathway for full coverage of a segmented region of interest (ROI; upper right)

**FIGURE 8:**
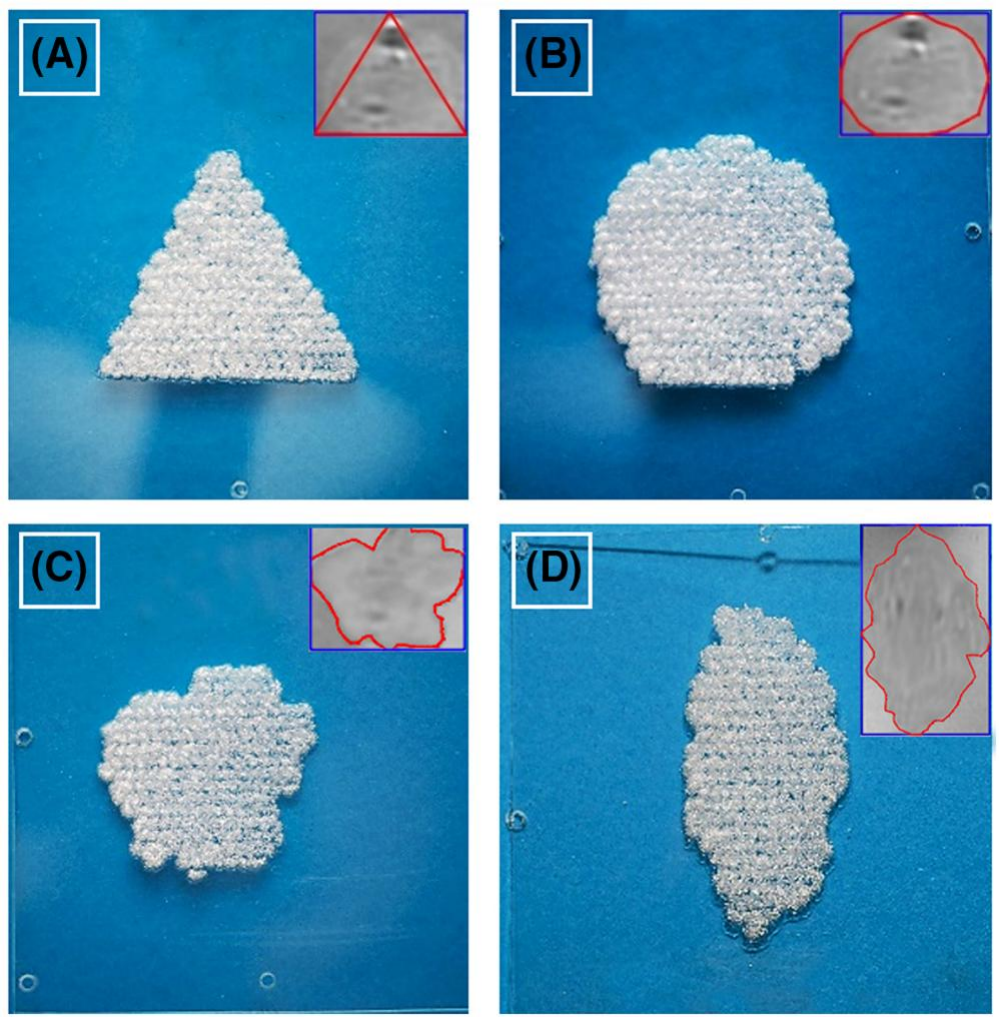
Ablation patterns on plastic films as planned on MR images of a tissue‐mimicking phantom (TMP; upper right of each image). The sonications were performed using acoustic power of 30 W for 7 s at each spot with a spatial step of 2 mm and a time delay of 30 s, using a single element transducer with central frequency of 2.75 MHz, radius of curvature of 65 mm, and diameter of 50 mm

Next, MR images were used for assessing the performance of the developed algorithm in accurate path planning. Figure 7 shows a two‐dimensional representation of sonication spots sorted along the planned Zig‐Zag pathway and the corresponding segmented ROI from an MR TMP image. Indicative results of ablation patterns produced on acrylic films are presented in Figure 8. A 2‐mm step was proven suitable for creating overlapping lesions when using the specific sonication protocol (30 W acoustical power and 7 s sonication time). As revealed by Figure 8, the shape of the ablated areas matches well with the corresponding ROIs.

## 4 DISCUSSION

In this study, the software of an already existing MRgFUS system was enhanced by implementing a full coverage path planning algorithm. Medical images are directly transfer from the MRI scanner to the software's interface for treatment planning. Briefly, the algorithm enables ROI segmentation on preoperative MR images and automatic generation of the sonication pathway based on a Zig‐Zag pattern. In fact, the marked area is filled with sonications spots that are being executed individually with a spatial step that is defined by the user. The estimated prediction time of the navigation path on MR images of 512 × 512 pixels is less 1 s for any lesion size, even when using a very small spatial step of 1 mm.

Thin acrylic films served as the main tool in the evaluation process. Initially, the power field of the FUS transducer was evaluated through visual and quantitative assessment of lesions produced on the plastic films at various distances from the transducer's surface. The results confirmed sufficient emission of ultrasonic energy and revealed a shift of the focal depth of about 10 mm towards the transducer. This information was useful for the next experiments. It should be also noted that lesion formation resulted from reflection at the plastic/air interface.

The main task in the evaluation process concerned the assessment of the path planning performance of the developed algorithm using a series of MR images. Successful planning was observed in all cases without any software defects. Next, the planned sonication protocols were executed by sonicating acrylic films using the integrated MRgFUS robotic system. Acrylic softens gradually as temperature rises. As an amorphous polymer, it crystallises and turns white when experiencing stress. In the current study, it was observed that heating at temperatures of about 55°C softens the material, and as a result acoustic pressure causes formation of white bumps referred to as lesions. Application of 30 W for 7 s (∼200 J energy) produced such temperatures, thus leading to lesion formation. The 2 mm step (with a 30 s delay) was proven suitable for creating overlapping lesions, whereas bigger steps resulted in discrete lesions. Well defined areas of overlapping lesions were produced on the films, with excellent match between the planned and experimental lesion shapes. High accuracy of robotic positioning of the source was also evidenced.

It is interesting to note that plastic films can be considered the cheapest phantom for quality assurance of hardware and software FUS systems. Notably, grid ablation on plastic films was previously proposed as a simple method to test the accuracy of robotic motion of FUS devices,^25^ being beneficial over other proposed MRI‐based methods^26^ in terms of cost‐effectiveness, ease of implementation, and accuracy.

Thermal diffusion from neighbouring spots is an important aspect in the therapeutic outcome of FUS treatment since it affects the formation of uniform lesions in tissue.^14^, ^15^ Production of asymmetric lesions was observed in optically transparent TMPs and excised tissue.^14^, ^15^ Curiel et al.^15^ reported a progressive enlargement of lesion size on in vitro pig liver after sonications in a line grid where each spot was exposed at acoustic power of 37 W for 2 s with 8 s waiting time between them. In the current study, although higher energy was applied at each spot (30 W for 7 s) with a similar spatial and temporal delay, there was no evidence of thermal diffusion effects on lesion production, meaning that visual assessment did not reveal significant variability in lesion density that would indicate variability in size of individual lesions. Note that uniformity of lesions is better observed in Figure 5 that shows a discrete pattern of almost equally spaced lesions of similar size and shape. This is expected given the smaller rate of heat transfer in acrylic plastic compared to soft tissue. In fact, the thermal conductivity of body organs is around 0.5–0.6 W/m‐K,^27^ whereas a value of 0.2 W/m‐K is reported literally for acrylic plastic.^28^ Accordingly, although phantoms with tissue like properties are especially useful in the evaluation of thermal protocols,^29^, ^30^ plastic films constitute a more practical and cost‐effective solution for assessing the performance of planning algorithms without thermal diffusion or other phantom‐dependent parameters affecting the results.

The previous version of the software included commands for controlling robotic motion and ultrasonic exposures. Advantageously, the developed algorithm enabled accurate path planning for full coverage of segmented ROIs of any shape, whereas the previous version allowed just for automating motion in XY rectangular grids.^31^ Such simplified motion algorithms were widely used in order to test the functionality of MRgFUS robotic devices in creating discrete and overlapping lesions, specifically by performing multiple ablations in line and square grid patterns in gel TMPs and animal tissue.^15^, ^32^, ^33^, ^34^, ^35^, ^36^

In the current version, the software can be interfaced with the MRI system enabling real time transfer and display of MR images on the software screen. The incorporation of path planning and MR thermometry tools aims to offer an efficient procedural workflow from the path planning stage to treatment plan execution. During treatment in the MRI setting, execution of the planned path will be visualised on the screen with simultaneous monitoring of thermal heating and feedback on ablation through the use of MR thermometry.

Follow up studies will test the performance of the developed software in *ex‐vivo* and in vivo animal tissue where thermal diffusion phenomena are more likely to affect lesion formation. This will enable better assessment of the performance of the Zig Zag pattern and will provide insights on the sonication protocol for safe in vivo studies. Generally, elevation of tissue temperature to more than 60°C for 1 s was proven to cause instantaneous death of cells mainly via coagulation mechanisms.^2^ In this regard, the optimal combination of acoustic parameters, motion step and time delay for achieving such ablative temperatures and formation of uniform lesions in soft tissue should be determined.

Although efforts were made for eliminating manual intervention and related errors in HIFU therapy planning,^7^, ^12^ there is still a need for fully automated procedures of high accuracy. The developed algorithm requires manual intervention for tumour segmentation through the user interface. The introduction of deep learning methods for automatic segmentation along the tumour margins is deemed necessary for achieving the precision required for clinical use. Such methods for automatic ROI localization on MR images are available in the literature^10^ and may be implemented in the algorithm in a later stage to eliminate user's involvement. Another advancement to be made is the transition from two‐dimensional to three‐dimensional planning, which may also require the incorporation of deep‐learning methods.

## 5 CONCLUSIONS

Overall, the herein proposed algorithm enables fast full coverage path planning on preoperative MR images. The developed algorithm as implemented in the software of an MRgFUS system was proven efficient in planning and executing ablation of two‐dimensional ROIs of complex geometry by sonicating transparent acrylic films in a Zig‐Zag pattern. Visual assessment revealed excellent match between the planned and experimental lesion shapes. Remarkably, plastic films probably constitute the cheapest phantom for quality assurance purposes of FUS devices. There are still challenges to be overcome for increasing the reliability of the developed algorithm through the development of a fully automated segmentation procedure.

## CONFLICT OF INTEREST

The authors have no conflict of interest.

## AUTHOR CONTRIBUTION

Andreas Georgiou contributed to the development of the path planning algorithm. Anastasia Antoniou contributed to the draughting of manuscript and scientific methods. Nikolas Evripidou contributed to the evaluation experiments. Christakis Damianou supervised the overall study, as well as the draughting of the manuscript.

## Data Availability

The data that support the findings of this study are available from the corresponding author upon reasonable request.
